# Influence of Cultivar and Biocontrol Treatments on the Effect of Olive Stem Extracts on the Viability of *Verticillium dahliae* Conidia

**DOI:** 10.3390/plants11040554

**Published:** 2022-02-20

**Authors:** Ana López-Moral, Carlos Agustí-Brisach, Francisco M. Leiva-Egea, Antonio Trapero

**Affiliations:** Departamento de Agronomía, María de Maeztu Unit of Excellence 2020-23, Campus de Rabanales, Universidad de Córdoba, Edif. C4, 14071 Córdoba, Spain; b92lomoa@uco.es (A.L.-M.); cagusti@uco.es (C.A.-B.); g42leegf@uco.es (F.M.L.-E.)

**Keywords:** biocontrol, endosphere, *Olea europaea*, plant–pathogen interactions, vascular pathogen, Verticillium wilt

## Abstract

The effect of olive (*Olea europaea*) stem extract (OSE) on the viability of conidia of *Verticillium dahliae*, the causal agent of Verticillium wilt of olive (VWO), is not yet well understood. Thus, the aim of this study was to determine the influence of the olive genotype (cultivar resistance) and the interaction between olive cultivars and biocontrol treatments on the effect of OSE on conidial germination of *V. dahliae* by *in vitro* sensitivity tests. To this end, OSE from cultivars Frantoio, Arbequina, and Picual, respectively tolerant, moderately susceptible, and highly susceptible to *V. dahliae*, were tested alone or after treatments with biological control agents (BCAs) and commercial products efficient at reducing the progress of VWO. *Aureobasidium pullulans* strain AP08, *Phoma* sp. strain ColPat-375, and *Bacillus amyloliquefaciens* strain PAB-24 were considered as BCAs. Aluminium lignosulfonate (IDAI Brotaverd^®^), copper phosphite (Phoscuprico^®^), potassium phosphite (Naturfos^®^), and salicylic acid were selected as commercial products. Our results indicate that the influence of biological treatments against the pathogen depends on the genotype, since the higher the resistance of the cultivar, the lower the effect of the treatments on the ability of OSE to inhibit the germination of conidia. In ‘Picual’, the BCA *B. amyloliquefaciens* PAB024 and copper phosphite were the most effective treatments in inhibiting conidia germination by the OSE. This work represents a first approach to elucidate the role of cultivar and biological treatments in modifying the effect on the pathogen of the endosphere content of olive plants.

## 1. Introduction

Verticillium wilt of olive (*Olea europaea* subsp. *europaea*; VWO) causes high levels of tree mortality and reduces fruit yield in most olive-growing areas worldwide and is considered the main limiting factor of olive in Mediterranean-type climate regions [1,2,3]. In southern Spain, the disease is one of the major concerns for olive growers. Although the global disease incidence in this region is around 0.5%, it can reach values higher than 20%, together with high levels of disease severity and tree mortality in certain areas across the Guadalquivir valley. The causal agent of VWO is the hemibiotrophic soil-borne fungus *Verticillium dahliae*, from which two populations, defoliating (D) and nondefoliating (ND) pathotypes, have been identified in olive, with D pathotype causing the most severe damage [1,4]. The pathogen develops microsclerotia (MS), which are dormant structures that not only confer the ability to survive in the soil for a long time, but also serve as the primary inoculum source in natural infections [1,2,4]. Regarding the life cycle of *V. dahliae*, MS germinate by the stimuli of the root exudates from the susceptible hosts, giving rise to infectious hyphae that penetrate the plant roots and grow until they reach the vascular system. Then, the xylem vessels of the infected plants are colonized by the pathogen through mycelia and conidia development, contributing to the occlusion of the vascular system, as well as the production of gels and tyloses in the xylem vessels. Altogether cause a reduction in water flow, leading to water stress, and, consequently, plants become wilted and eventually die [5,6,7].

The innate biology of the pathogen, besides of the agronomical factors related to the intensification of olive crop, favours a year-by-year increase in disease incidence and have made it difficult to control VWO, making this disease one of the largest threats to olive groves worldwide [1,8,9]. Likewise, no truly efficient method to control VWO has been reported. Thus, there is no doubt that an integrated disease management (IDM) strategy is needed to prevent *V. dahliae* infections by both pre- and post-planting control measures in olive groves [1,2,10]. 

Considering IDM against VWO, both genetic resistance and biological control methods must be combined to achieve synergistic effectiveness to reduce both pathogen dispersal and disease incidence in the field. It is well known that disease severity varies depending on the olive cultivars, with the selection of cultivar being essential to avoid serious infections in the field. For instance, ‘Picual’ is considered one of the most susceptible olive cultivars, whereas ‘Arbequina’ and ‘Hojiblanca’ have shown moderate susceptibility, and ‘Changlot Real’, ‘Empeltre’, and Frantoio have high levels of tolerance [11,12]. On the other hand, important advances in biological control against VWO have been achieved in the last two decades [2,13]. In this regard, a broad diversity of essential oils, organic amendments, and biological control agents (BCAs), including endophytic bacteria and fungi, plant biostimulants, and host resistance inducers, have been evaluated both under controlled and natural field conditions towards selection of the best candidates for the control of VWO [3,14,15,16,17,18]. Foliar or root applications with two beneficial microorganisms, the fungus *Aureobasidium pullulans* AP08 and the bacterium *Bacillus amyloliquefaciens* PAB-024; and two phosphonate salts, one of copper and the other of potassium, were effective in reducing disease progression in artificially inoculated olive plants [3]. 

Due to the different behaviour patterns previously observed on the effect of all these treatments against VWO, their mechanisms of action have been explored, elucidating both direct effects, such as dual culture assays and *in vitro* sensitivity tests [3], and indirect effects, such as the effect on root exudates inhibiting MS and conidia germination enhancing the natural plant defence response [7,19] on the viability of the infectious structures of *V. dahliae* and VWO progress. These previous studies contributed to better understanding of how these biocontrol products can act against the pathogen; however, knowledge generated here opens new paths to be explored concerning such a topic. In this way, determining whether these products can modulate the effect of endosphere contents of the treated olive plants on *V. dahliae* infection would suppose other relevant knowledge towards to better elucidating their mode of action. 

The xylem vessels are an ideal niche for microbial endophytes such as the Verticillium wilt pathogen by providing an effective internal pathway for whole-plant colonization and by acquiring the scarce nutrients available in xylem sap, either by enzymatic digestion of host cell walls, by invading neighbouring cells, or by inducing leakage of nutrients from surrounding tissues [20,21]. Indeed, xylem sap contains a wide range of compounds beyond water and minerals, such as amino acids [22], organic acids [23], and vitamins [24]. The xylem sap composition of woody plant species, including olive tree, has been characterized in several previous studies [25]. However, this composition can be influenced by multiple factors, such as the water content of the soil [26]; the cultivar; the type and age of organs selected; and the incidence of microbial interactions, including infection by plant pathogens, among others [25]. Related to this aspect, recently, Anguita-Maeso et al. [27] determined that the xylem microbiome of olive plants inoculated with *V. dalhiae* increases the diversity of bacterial communities compared to non-inoculated plants. In addition, these same authors also showed a breakdown of resistance to *V. dahliae* in wild olive trees related to a modification of their xylem microbiome [27]. However, to our knowledge, there are no scientific studies that address the effect of the content of the olive endosphere, including xylem sap, on the viability of infectious structures of *V. dahliae*. Thus, the aim of this study was to determine the influence of the olive genotype (cultivar resistance) and the interaction between cultivars and biocontrol treatments on the effect of olive stem extract (OSE) on the viability of conidia of *V. dahliae* *in vitro*. 

## 2. Results

### 2.1. Effect of Olive Stem Extract on Conidial Viability of Verticillium dahliae

#### 2.1.1. Experiment I: Effect of Olive Cultivars

**Figure 1:**
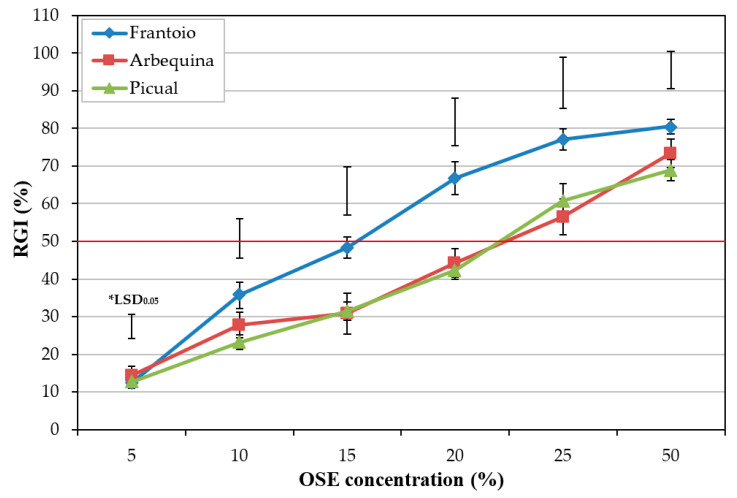
Effect of olive stem extract (OSE) from healthy olive plants of cvs. Frantoio (tolerant), Arbequina (moderately susceptible), and Picual (highly susceptible) on relative conidial germination inhibition (RGI) of *Verticillium dahliae* isolate V180. Data are the means of 240 conidia (six replicates) per combination of olive cultivar and OSE concentration. Vertical bars represent the standard error of the means. *LSD bars represent the critical values for comparison at *P* = 0.05.

The results on the effect of OSE from plants of cvs. Frantoio (tolerant), Arbequina (moderately susceptible), and Picual (highly susceptible) at 5, 10, 15, 20, 25, and 50% concentration on the conidial viability of *V. dahliae* showed that the higher OSE concentration, the higher the RGI. Marked differences between the three olive cultivars were detected from OSE at 10%, whereas that from ‘Frantoio’ showed higher RGI values than that from ‘Arbequina’ and ‘Picual’; however, in these last two cases, the inhibition did not differ significantly. At the highest concentration evaluated (OSE at 50%), RGI values ranged from 68.9 ± 2.9 to 80.5 ± 1.9% for ‘Picual’ and ‘Frantoio’, respectively (Figure 1).

In addition, significant differences were observed for EC_50_ between cultivars (*P* = 0.0239). The OSE from ‘Frantoio’ showed the lowest EC_50_ value (EC_50_ = 16.2 ± 0.88) compared to the OSE from the rest of cultivars, whereas OSE from ‘Arbequina’ (EC_50_ = 21.7 ± 1.15) and ‘Picual’ (EC_50_ = 20.4 ± 1.08) did not differ significantly from one another (Table 1).

#### 2.1.2. Experiment II: Influence of Treatments

**Figure 2:**
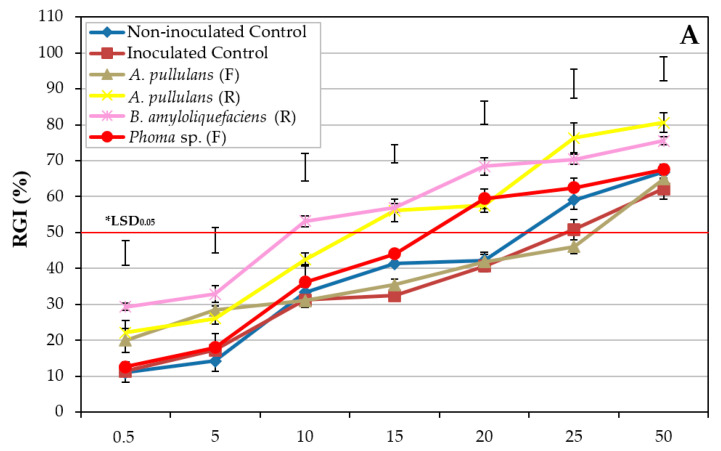

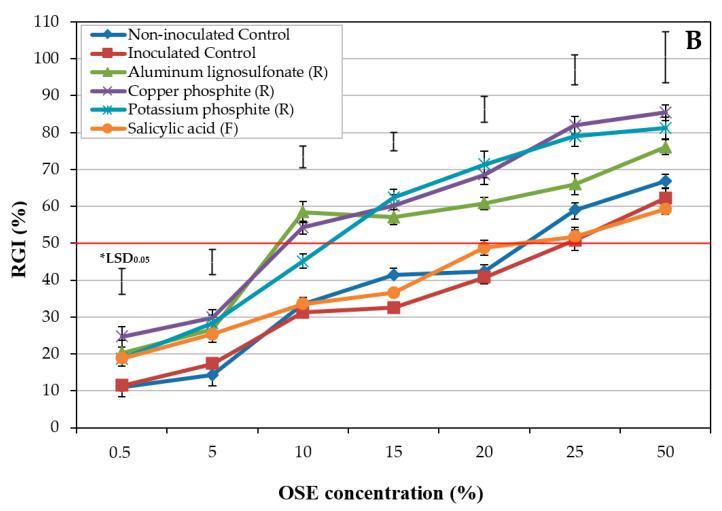
Effect of olive stem extract (OSE) from healthy olive plants of cv. Picual treated by foliar (F) or root (R) applications with (**A**) microorganisms (biological control agents) and (**B**) commercial products on the relative conidial germination inhibition (RGI) of *Verticillium dahliae* isolate V180. In each graph, data are the means of 240 conidia (six replicates) per combination of compound and OSE concentration. Vertical bars represent the standard error of the means. *LSD bars represent the critical values for comparison at *P* = 0.05.

The influence of the OSE from olive plants of cv. Picual treated with different treatments on conidial viability showed great differences between treatments (Figure 2). In general, the OSE from plants treated with commercial products showed higher RGI values than those from OSE obtained from plants treated with BCAs. Only the OSE from plants treated by root applications with *A. pullulans* AP08, aluminium lignosulfonate, and copper phosphite showed RGI values higher than 80% when they were tested at the highest concentration. 

There were significant differences for EC_50_ between treatments (*P* < 0.0001). In general, OSE from treated plants showed significantly lower EC_50_ values than that from both negative and positive control (EC_50_ = 21.5 ± 1.43 and 24.9 ± 0.69, respectively), with the exception of OSE from plants treated by spraying with *A. pullulans* AP08 (EC_50_ = 24.7 ± 1.61), *Phoma* sp. (EC_50_ = 17.3 ± 0.55), and salicylic acid (EC_50_ = 22.1 ± 2.05), which did not differ from the controls (Table 2).

#### 2.1.3. Experiment III: Interaction between Olive Cultivar and Treatments

**Figure 3:**
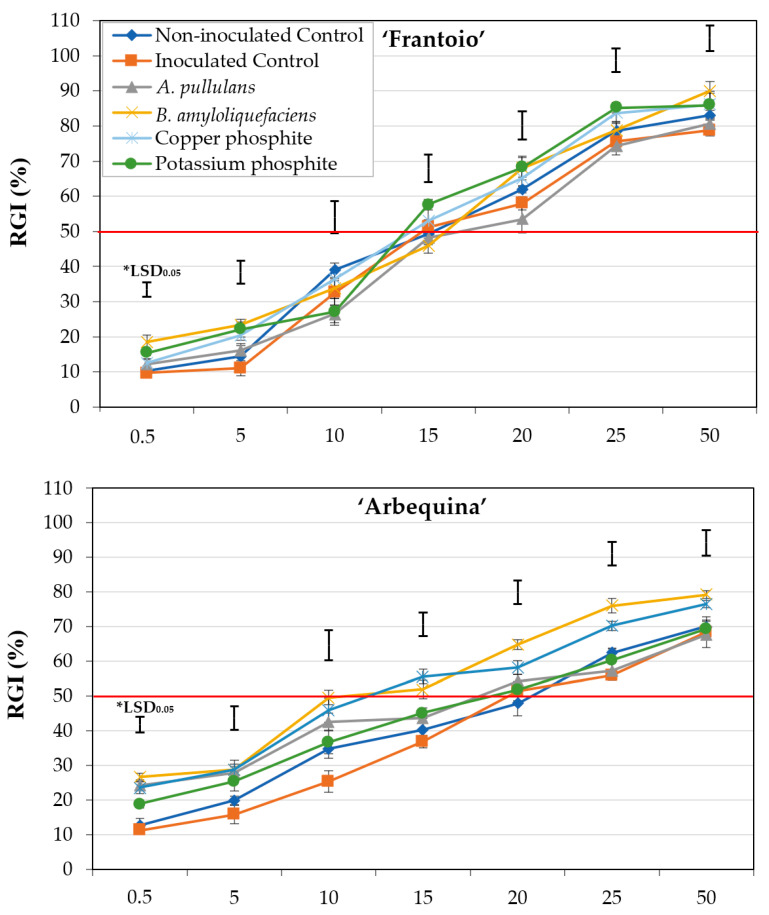

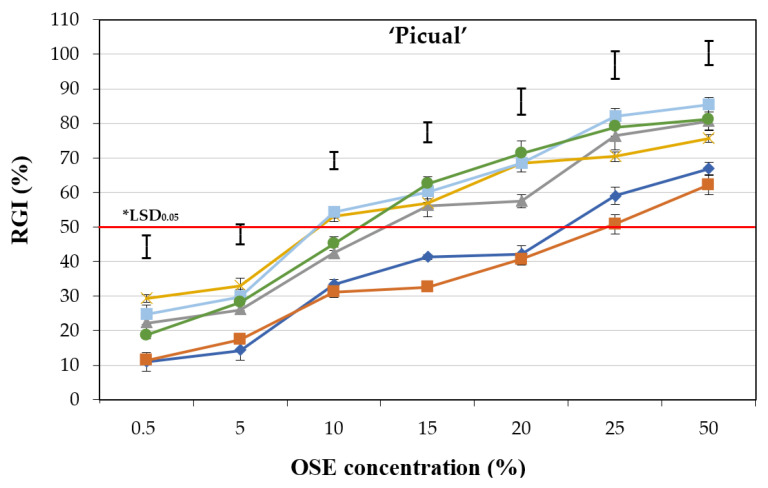
Effect of olive stem extract (OSE) from healthy olive plants of cvs. Frantoio, Arbequina, and Picual treated by root applications with the most effective compounds from *Experiment II* (*Aureobasidium pullulans*, *Bacillus amyloliquefaciens*, copper phosphite, or potassium phosphite) and from non-treated control plants, both non-inoculated and inoculated with *Verticillium dahliae* isolate V-180 on the relative conidial germination inhibition (RGI) of *V. dahliae* isolate V180. In each graph, data are the means of 240 conidia (six replicates) per combination of olive cultivar and OSE concentration. Vertical bars represent the standard error of the means. *LSD bars represent the critical values for comparison at *P* = 0.05.

OSE from plants of cv. Frantoio did not show marked differences in RGI between treatments. A moderate effect on RGI was observed for OSE from treated plants of cv. Arbequina, with RGI slightly increasing for OSE from plants treated with *A. pullulans* and copper phosphite. On the other hand, significant differences in RGI were observed in ‘Picual’ between the OSE from treated and control plants, as well as between the OSE from plants treated with the different BCAs and chemical products (Figure 3).

ANOVA confirmed that there were no significant differences for EC_50_ between treated and non-treated plants of cv. Frantoio (*P* = 0.9510). In ‘Arbequina’, significant differences for EC_50_ were only observed between the OSE from plants treated with *B. amyloliquefaciens* (EC_50_ = 13.0 ± 1.57) and copper phosphite (EC_50_ = 14.2 ± 1.19) compared to that obtained for OSE of the controls. Finally, in ‘Picual’, the OSE from treated plants showed significantly lower EC_50_ values than those of the OSE from both negative and positive controls; however, EC_50_ did not differ significantly between the different products evaluated (Table 3).

## 3. Discussion

Although recent studies have revealed that *V. dahliae* modulates the xylem microbiome in olive plants by increasing the diversity of bacterial communities when the pathogen is present in the soil [27], the influence that olive cultivar, biological treatments, and their interaction could have on the endosphere against the viability of the infectious structures of *V. dahliae* has not yet been reported. Thus, this work represents a first approach to elucidate the role of cultivar and biological treatments in modifying the effect of the endosphere contents on the pathogen in olive plants.

In this study, 6-month-old potted olive plants were used because, based on our extensive experience working with the olive tree and *V. dahliae* pathosystem, it is not only the age limit to reproduce the symptoms of the disease by artificial inoculation of plants in small pots [3,14], but also considering the limitations that the size and physiological structure of plant tissues could have in the extraction of contents from the endosphere by Cadahía’s method [28]. Regarding this last aspect, we have to consider that Cadahía calls ‘sap’ to the total liquid extract from the endosphere (called OSE in this study) that comes from both the xylem and phloem of the plant. The ideal would probably be to obtain raw sap (xylem) and elaborated sap (phloem) separately to evaluate the effect of the raw sap alone, but despite this limitation, we chose this method instead of the sap-extraction method using a Scholander chamber [29] for the following reasons: (i) because it has been demonstrated that the extract of pure sap from the xylem of olive plants contains a wide diversity of bacterial communities [27], which could mask the effect of OSE on the germination of conidia in *in vitro* sensitivity tests; (ii) consequently to this first reason, the extraction of OSE using Cadahía’s method guaranties the absence of living microorganisms, as well as cellular debris in the extract, since the plant material is subjected to consecutive immersion and freezing treatments in diethyl ether during the extraction process; and (iii) because it has been recognized as reliable method to determine the nutritional levels of plants, since results using ‘sap-like’ extracts (OSE) contrast well with those of the yield and quality of the harvest of several fruit crops, including the olive tree, in more than 40 years of experience [28].

Only the effect of OSE on the germination of conidia was evaluated in this study because conidia are the infectious structures of *V. dahliae* directly affected by the sap, considering the life cycle of the pathogen, i.e., the pathogen infects plants through the root by the germinated MS and then systemically colonizes the infected plants by producing conidia in the xylem vessels [1,6]. In addition, the treatment combinations (treatments and/or mode of application) evaluated in this study were selected because they all resulted in high effectiveness in inhibiting the viability of the infectious structures of *V. dahliae*, as well as reducing the progress of the disease in previous studies by López-Moral et al. [3].

Our results revealed that OSE from ‘Frantoio’ (tolerant) showed higher RGI values than that from ‘Arbequina’ (moderately susceptible) and ‘Picual’ (highly susceptible), whereas the inhibition did not differ markedly between the last two cultivars (*Experiment I*). In ‘Picual’, the influence of OSE from the treated plants on the inhibition of conidia germination varied significantly between the evaluated treatments. In this cultivar, the BCA *B. amyloliquefaciens* PAB-024, and a phosphonate salt [copper phosphite (Phoscuprico^®^)] were the most effective in inhibiting conidia germination by the OSE (*Experiment II*). In addition, when the four selected treatments were applied to the three olive cultivars, their influence on the effect of OSE on the inhibition of conidia germination was not significant between treatments for ‘Frantoio’, whereas moderate and markedly significant differences between treatments were observed for ‘Arbequina’ and ‘Picual’, respectively (*Experiment III*). Finally, although some differences can be observed between both positive and negative controls in RGI as the OSE concentration increases (Figure 2 and Figure 3), the EC_50_ data did not show significant differences between either control in any case. Therefore, these data suggest that the biotic stress caused by the infection of the pathogen in the plant does not influence the effect of OSE on the conidial germination of *V. dahliae*. 

As a first conclusion, our results indicate that the influence of biological treatments against the pathogen depends on the genotype, since the greater the resistance of the cultivar, the lower the influence of the treatments on the ability of OSE to inhibit conidia germination. Thus, the results suggest that the high tolerance to *V. dahliae* conferred by the ‘Frantoio’ genotype prevails over the treatments, even those that were more effective against the pathogen in susceptible cultivars. These results are in agreement with those obtained recently by López-Moral et al. [7], who determined the influence of cultivars and biological treatments on the effect of root exudates from olive plants on the viability of MS and conidia of *V. dahliae*. These authors demonstrated that root exudates induced germination of conidia and MS of *V. dahliae* and that the genotype significantly affected this ability; the root exudates from ‘Frantoio’ did not show a significant effect on the induction of MS and conidia germination compared to the control, whereas those from ‘Arbequina’ and ‘Picual’ showed a moderate and marked effect, inducing the viability of both MS and conidia of *V. dahliae*, respectively [7]. 

Regarding genetic resistance, our study reveals new knowledge about the relationship that the sap and the olive cultivar could have, favouring or interfering with the colonization of the xylem by the pathogen. However, in order to elucidate how the cultivar factor could influence the effect of its sap on the colonization of the pathogen, further interaction studies with the xylem anatomy of each cultivar should be conducted in the future. In fact, previous studies that evaluated the anatomy of the xylem of healthy olive trees of cvs. Frantoio and Picual showed significant differences not only in the parameters related to water transport but also in the density of vessels associated with a larger or smaller conduction area in the xylem tissue, both parameters being significantly higher in ‘Frantoio’ than in ‘Picual’ [30].

Regarding the influence of the treatments on the effect of OSE on the viability of the conidia, our results are also in agreement with those obtained by López-Moral et al. [7]. These authors revealed that the root exudates from plants of the three cultivars (Frantoio, Arbequina, and Picual) treated with the same four treatments evaluated in *Experiment III* of the present study showed significant differences in their effect on MS and conidia germination, and the genotype also significantly affected this ability. In this case, the treatment with *A. pullulans* AP08 was most effective, showing a significant effect inhibiting conidia germination in ‘Arbequina’ and MS germination in ‘Arbequina’ and ‘Picual’, but non-significant effect in these two parameters was observed in ‘Frantoio’. Regarding the rest of the evaluated treatments, the root exudates from plants treated with copper phosphite, potassium phosphite, and *B. amyloliquefaciens* PAB-24 gave rise to a significant inhibition in the germination of conidia or MS but only in cv. Arbequina [7]. In addition, previous studies conducted by López-Moral et al. [19] to evaluate these treatments as potential inducers of host resistance against VWO showed that the two BCAs *A. pullulans* AP08 and *B. amyloliquefaciens* PAB-024, as well as the phosphonate salt Phoscuprico^®^, had the ability to accumulate jasmonic acid (JA) and JA-isoleucine in leaves, stem, or roots of treated olive plants of cv. Picual. These results also suggest an implication of JA in the host resistance induced by these treatments. This last aspect could be directly related to our results, since it is well known that xylem infections by vascular pathogens cause drastic metabolic changes in the cells of the xylem parenchyma adjacent to the infected vessels. These metabolic changes lead to the accumulation of different proteins and secondary metabolites in xylem sap during pathogen colonization, including pathogenesis-related proteins (PR proteins), enzymes (e.g., peroxidases, proteases, xyloglucan-endotransglycosylase, xyloglucan-specific endoglucanase protein inhibitor), phenols, phytoalexins, and lignin, which help to enhance the natural defence mechanisms of the plant [31,32,33,34,35]. On the other hand, due to the fact that the OSE did not have a live microbiome as a consequence of the extraction method used, studies to determine whether the treatments applied in this work could influence the modification of the xylem microbiome of olive plants must be conducted. In this way, recent studies performed by Anguita-Maeso et al. [27] determined that the xylem microbiome of olive plants inoculated with *V. dahliae* increases the diversity of bacterial communities compared to non-inoculated plants. However, how the xylem microbiome could be modified by biological treatments favouring *V. dahliae* inhibition is still uncertain.

In summary, the method used in this study to obtain endosphere contents of olive plants, called OSE or ‘sap-like’, for further analysis in the laboratory against *V. dahliae* can be considered valid and useful, since all our results agree with those obtained in previous studies [3,7,19]. The knowledge generated here represents a first approach in the study of how genotype and/or biological treatments can influence the extracts of olive plants by inhibiting the germination of conidia or of MS of *V. dahliae*. This knowledge could be useful in the future to prevent infections or mitigate the progression of disease within the framework of the current ‘from-farm-to-fork’ strategy to obtain safe and healthy fruits. 

## 4. Materials and Methods

### 4.1. Plant Material

Healthy 6-month-old rooted cuttings of three olive cultivars representative of different degrees of susceptibility to *V. dahliae* were used: ‘Frantoio’ (tolerant), ‘Arbequina’ (moderately susceptible), and ‘Picual’ (highly susceptible) [11,12]. The plants were obtained from a commercial nursery and were grown in peat moss in plastic pots (0.5 L). They were pre-conditioned in a controlled growth chamber (22 ± 2 °C, with a 14:10 h (light:dark) photoperiod of white fluorescent light (10,000 lux) and 60% relative humidity (RH)) for 1 month to induce active growth. During this month, plants were irrigated three times per week with 350 mL of water per plant. 

### 4.2. Fungal Strain and Inoculum Preparation

The *V. dahliae* isolate V180 was used in all the experiments of this study [3]. It was stored as single-spore isolate on potato dextrose agar (PDA; Difco^®^ Laboratories, MD, USA) slants filled with sterile paraffin oil at 4 °C in the dark in the collection of the Department of Agronomy at the University of Córdoba (DAUCO, Spain). Before conducting each experiment, small mycelial fragments of the colonized agar from the tube were plated onto PDA acidified with 2.5 mL L^−1^ lactic acid (APDA) and incubated at 24 °C in the dark for 10 days in order to obtain fresh colonies. Then, they were transferred to PDA and incubated as previously described. 

### 4.3. Effect of Olive Stem Extract (OSE) on Conidial Viability of Verticillium dahliae

#### 4.3.1. Obtaining Stem Extract 

For obtain OSE, the main stem of the plants was cut at its base, and the entire main stem and shoots were used. Leaves and roots were discarded. Subsequently, most of the cortical tissue of stems and shoots was removed manually using sandpaper, and the peeled stems were sprayed with distilled water and kept at 4 °C in the dark to avoid desiccation until further processing. OSE was obtained by the analytical laboratory ‘C+E Analítica’ (San José de la Rinconada, Seville, Spain) following the protocol described by Cadahía [28]. Specifically, once in the analytical laboratory, the shoots and stems were cut into 0.5 cm fractions, immersed in ethyl ether, and kept at −20 °C for 2 h. As a consequence of the freezer step, the water contained in the plant tissues crystallised, breaking the cell walls, which later allowed a sap-like extract to be obtained. At the same time, the ether is able to extract the chlorophyll that could interfere with the analytical process. After this step, the plant material was defrosted, and the aqueous phase (endosphere contents) was separated from the ether to obtain OSE by means of a hydraulic press [28]. 

#### 4.3.2. Experiment I: Effect of Olive Cultivars

To evaluate the effect of OSE from different olive cultivars on conidia viability, plants of cvs. Frantoio, Arbequina, and Picual were used. They were maintained for one month in growth chambers, as described above. For this period, they were arranged in a randomized complete block design with three blocks and four replicated plants per cultivar (3 × 4 = 12 plants per cultivar; 36 plants in total). The OSE was obtained by joining all the plants of each block, so there were three experimental units of OSE (≈20 mL) per cultivar. 

#### 4.3.3. Experiment II: Influence of Treatments

To evaluate the influence of different treatments on the effect of OSE on *V. dahliae*, olive plants of cv. Picual were treated with seven treatments, including three BCAs and four commercial products (Table 4). These treatments and the type of application (foliar and/or irrigation) were selected for this study because of their significant efficacy against *V. dahliae in vitro*, as well as against the progress of VWO in planta demonstrated in previous studies [3].

Plants were treated with the water solution or suspension of each commercial product or BCA (Table 4), respectively, at 6-, 5-, and 3 weeks before obtaining OSE. Additionally, non-treated but inoculated plants by cornmeal–sand mixture (CSM; sand, cornmeal and distilled water; 9:1:2, weight: weight:volume) colonized by *V. dahliae* isolate V180 (theoretical inoculum density of the final substrate = 10^7^ CFU g^−1^) were included as a positive control; and non-treated and non-inoculated plants as negative control. Plant inoculation was conducted 4 weeks before obtaining the OSE. Treatments or plant inoculation were conducted following the protocols described by López-Moral et al. [3]. A randomized complete block design with three blocks and four replicated olive plants per treatment (*n* = 10; eight treatments and two controls) was used (120 plants in total). 

This experiment was maintained for six weeks after the first treatment was applied. Subsequently, OSE was obtained from plants of each treatment, as well as from plants of both positive and negative control, joining all the plants of each block, i.e., there were three experimental units of OSE (≈20 mL) per treatment or control.

#### 4.3.4. Experiment III: Interaction between Olive Cultivars and Treatments

To evaluate the influence of the interaction between olive cultivars and treatments on the effect of OSE on *V. dahliae*, olive plants of the three cvs. described above were treated with two BCAs (fungus *Aureobasidium pullulans* strain AP08 and bacterium *Bacillus amyloliquefaciens* strain PAB-24) and two commercial products [copper phosphite (Phoscuprico^®^) and potassium phosphite (Naturfos^®^)] (Table 4). These treatments were selected for this experiment for their efficacy in inhibiting conidial germination of *V. dahliae* in *Experiment II*. 

Plants were treated 6-, 5-, and 3 weeks before obtaining the OSE and inoculated 4 weeks before obtaining the OSE. The treatments, OSE extraction, and plant inoculation were conducted as described above. For each olive cultivar, a positive and a negative control were included, as described in *Experiment II*. A randomized complete block design with three blocks and four replicated olive plants per treatment (*n* = 6, four treatments and two controls) and cultivar (*n* = 3) combination was used (72 plants per olive cultivar; 216 plants in total). 

This experiment was maintained for six weeks after the first treatment was applied. Subsequently, OSE was obtained from plants of each treatment and olive cultivar combination, as well as from plants of the positive and negative control, joining all the plants of each block, so there were three experimental units of OSE (≈20 mL) per treatment or control and cultivar combination. 

#### 4.3.5. Conidia Viability *In Vitro*

For each set of experiments, conidial suspensions were obtained from 14-day-old colonies of *V. dahliae* isolate V180 growing on PDA, as described previously, and adjusted to 8 × 10^5^ conidia mL^−1^ using a haematocytometer. In parallel, OSE solutions were adjusted to 0, 1, 10, 20, 30, 40, 50, and 100% in sterile deionized distilled water (SDDW). Subsequently, a 5 μL drop of the conidial suspension was placed in the centre of a microscope coverslip (20 × 20 mm); then, a 5-μL drop of the OSE solution was mixed. Thus, the OSE was evaluated at the following final concentrations: 0, 0.5, 5, 10, 15, 20, 25, and 50%, with a concentration of 0% consisting of a 5 μL drop of the conidial suspension mixed with a 5 μL drop of sterile SDDW as a control. The coverslips were placed inside Petri dishes containing water agar, which were used as humid chambers, and were incubated at 23 ± 2 °C in the dark for 24 h. After the incubation period, a 5 µL drop of 0.01% acid fuchsine in lactoglycerol (1:2:1 lactic acid:glycerol:water) was added to each coverslip to stop conidial germination, and they were mounted on a slide. For each experiment (*I*, *II*, and *III*), there were three replicated coverslips per concentration of OSE obtained from each block and from each treatment or control (OSE at 0%, i.e., only SDDW). All the experiments were conducted twice.

In all cases, a total of 120 randomly selected conidia per replicated coverslip were observed at a ×400 magnification by means of a Nikon Eclipse 80i microscope (Nikon Corp., Tokyo, Japan), and the germinated and non-germinated conidia were counted. Conidia were considered germinated when the germ tube was at least one-half of the longitudinal axis of the conidia. Conidial viability was estimated as percentage (%) of conidial germination for each OSE concentration, and then, the inhibition of conidial germination (RGI; %) was estimated with respect to the control according to the following formula:

RGI (%) = [(Ge_control_ × Ge_OSEsolution_)/Ge_control_]

where Ge_control_ = percentage of germinated conidia after incubation in the SDDW, and Ge_OSEsolution_ = percentage of germinated conidia after incubation in the OSE solution from treated plants [36]. The RGI data were linearly regressed over the OSE concentration, and the predicted values of the effective OSE concentrations (μg mL^−1^) inhibiting 50% (EC_50_) of conidial germination were obtained from the fitted regressions.

#### 4.3.6. Data Analysis

Data of EC_50_ from the two repetitions of each experiment were combined after checking for homogeneity of the experimental error variances by the F test (*P* ≥ 0.05)p. Subsequently, data were tested for normality, homogeneity of variances, and residual patterns. For *Experiments I* and *II*, a one-way ANOVA was conducted, with the EC_50_ as dependent variable and ‘cultivar’ or ‘treatment’ as independent variables. For *Experiment III*, a factorial ANOVA was conducted with EC_50_ as dependent variable, and ‘cultivar’, ‘treatment’, and their interaction as independent variables. Since interaction was significant (*P* = 0.0001), one-way ANOVAs were conducted for each olive cultivar. Treatment means were compared according to Fisher’s protected LSD test or Tukey´s HSD test (both at *P* = 0.05) for the *Experiment I* (*n* = 3) and *III* (*n* = 6), or for *Experiment II* (*n* = 10), respectively [37]. Study data were analyzed using Statistix 10.0 software [38].

## 5. Conclusions

Our results indicate that the influence of biological treatments against the pathogen depends on the genotype, since the greater the resistance of the cultivar, the lower the influence of the treatments on the ability of OSE to inhibit conidia germination. In ‘Picual’, the most susceptible cultivar to VWO, the BCA *B. amyloliquefaciens* PAB-024 and a phosphonate salt [copper phosphite (Phoscuprico^®^)] were the most effective treatments in inhibiting conidia germination by OSE. Thus, the results suggest that the high tolerance to *V. dahliae* conferred by the ‘Frantoio’ genotype prevails over the treatments, even those that were more effective against the pathogen in susceptible cultivars. The method used in this study to obtain endosphere contents of olive plants, called OSE or ‘sap-like’ for further analysis in the laboratory against *V. dahliae* can be considered valid and useful since all our results agree with those obtained in previous studies. Thus, this work represents a first approach to elucidate the role of cultivar and biological treatments in modifying the effect on the pathogen of the endosphere content of olive plants.

## Figures and Tables

**Table 1:** Effective concentrations of olive stem extract (OSE) from cvs. Frantoio, Arbequina, and Picual to inhibit 50% of conidial germination (EC_50_; μL mL^−1^) of *Verticillium dahliae* isolate V180.

Cultivar	EC_50_ (μL mL^−1^) ^a^
Frantoio	16.2 ± 0.88 b
Arbequina	21.7 ± 1.15 a
Picual	20.4 ± 1.08 a

^a^ EC_50_ of conidial germination was calculated as predicted value of the linear regression of the relative germination inhibition (%) over OSE concentration. Values represent the average of 240 conidia (six replicates) per extract (cultivar) and OSE concentration. Means followed by a common letter do not differ significantly according to Fisher’s protected LSD test at *P* = 0.05.

**Table 2:** Effective concentrations of olive stem extract (OSE) from cv. Picual treated with several compounds to inhibit 50% of conidial germination (EC_50;_ μL mL^−1^) of *Verticillium dahliae* isolate V180.

Treatment ^a^	Application	EC_50_ (μL mL^−1^) ^b^
Control (−)		21.5 ± 1.43 ab
Control (+)		24.6 ± 0.69 a
Aluminum lignosulfonate	Root	12.1 ± 1.38 cd
*Aureobasidium pullulans*	Foliar	24.7 ± 1.61 a
*A. pullulans*	Root	13.1 ± 1.01 cd
*Bacillus amyloliquefaciens*	Root	10.3 ± 1.26 d
Copper phosphite	Root	10.1 ± 1.34 d
*Phoma* sp.	Foliar	17.3 ± 0.55 bc
Potassium phosphite	Root	11.8 ± 1.52 cd
Salicylic acid	Foliar	22.1 ± 2.05 ab

^a^ All compounds were applied by foliar or root applications in non-inoculated plants. Control (-): non-treated and non-inoculated plants; Control (+): non-treated and inoculated plants with *V. dahliae* isolate V-180. ^b^ EC_50_ of conidial germination was calculated as predicted value of the linear regression of the relative germination inhibition (%) over OSE concentration. Values represent the average of 240 conidia (six replicates) per extract (cultivar) and OSE concentration. Means followed by a common letter do not differ significantly according to Tukey’s HSD test (*P* = 0.05).

**Table 3:** Effective concentrations of olive stem extract (OSE) from cvs. Frantoio, Arbequina, and Picual and treated with the most effective compounds to inhibit 50% of conidial germination (EC_50;_ μL mL^−1^) of *Verticillium dahliae* isolate V180.

Treatment ^a^	EC_50_ (μL mL^−1^) ^b^		
	Frantoio	Arbequina	Picual
Control (−)	15.2 ± 2.07 a	20.1 ± 0.90 a	21.5 ± 0.85 a
Control (+)	16.1 ± 1.39 a	20.0 ± 2.44 a	24.6 ± 0.69 a
*Aureobasidium pullulans*	16.4 ± 1.36 a	17.9 ± 0.88 ab	13.1 ± 1.01 b
*Bacillus amyloliquefaciens*	14.8 ± 1.87 a	13.0 ± 1.57 c	10.3 ± 1.26 b
Copper phosphite	14.9 ± 0.83 a	14.2 ± 1.19 bc	10.1 ± 1.34 b
Potassium phosphite	14.7 ± 1.23 a	18.3 ± 0.92 ab	11.8 ± 1.52 b

^a^ All evaluated treatments were applied by root applications in non-inoculated plants. Control (−): non-treated and non-inoculated plants; Control (+): non-treated and inoculated plants with *V. dahliae* isolate V-180. ^b^ EC_50_ of conidial germination was calculated as predicted value of the regression: relative germination inhibition (%) over OSE concentration. Values represent the average of 240 conidia (six replicates) per extract (cultivar) and OSE concentration. Means followed by a common letter do not differ significantly according to Fisher’s protected LSD test at *P* = 0.05.

**Table 4:** Biological and chemical products evaluated in this study ^a^.

Active Ingredient(s)	Trade Name/Formulation ^b^	Manufacturer	Class (FRAC Code) ^c^	Dose ^d^	
				Foliar	Root
Biological Control Agents (BCAs) ^e^					
*Aureobasidium pullulans*	AP08	DAUCO ^d^	Fungal (NC)	10^6^ conidia mL^−1^	10^6^ conidia mL^−1^
*Bacillus amyloliquefaciens*	PAB-024	DAUCO	Bacterial (NC)	n/e	10^8^ CFU mL^−1^
*Phoma* sp.	ColPat-375	DAUCO	Fungal (NC)	10^6^ conidia mL^−1^	n/e
Chemical Products					
Aluminum lignosulfonate	IDAI Brotaverd^®^-EW	IDAI Nature	Inorganic salt (NC)	n/e ^f^	5 mL L^−1^
Copper phosphite	Phoscuprico^®^-EW	Agri nova Science	Phosphorous acid and salts (P07)	n/e	10 mL L^−1^
Potassium phosphite	Naturfos^®^-EW	Daymsa	Phosphorous acid and salts (P07)	n/e	8 mL L^−1^
Salicylic acid	Salicylic acid-SL	Sigma-Aldrich	Organic acid (NC)	5 mM (0.69 g L^−1^)	n/e

^a^ Products and type of application evaluated in the present study were selected for their efficacy against *V. dahliae* observed in the previous study conducted by López-Moral et al. [3]. ^b^ EW: emulsion, oil in water; SL: soluble concentrate. ^c^ Group and code numbers are assigned by the Fungicide Resistance Action Committee (FRAC) according to different modes of actions (NC: not classified; for more information, see http://www.frac.info/, accessed on 24 November 2021). ^d^ Maximum dose for foliar or root applications recommended for the manufacturers of the commercial compounds evaluated in this study. Fungal and bacterial inocula from the BCAs (AP08, PAB-024, and ColPat-375) were prepared and adjusted according to Varo et al. [14]. ^e^ All the BCAs used in this study are maintained in the collection of the Agroforestry Pathology Research Group at the Department of Agronomy, University of Córdoba (DAUCO), Spain. ^f^ n/e: non-evaluated products and dose combinations in this study.

## Data Availability

Not applicable.
